# Pcgf1 Regulates Early Neural Tube Development Through Histone Methylation in Zebrafish

**DOI:** 10.3389/fcell.2020.581636

**Published:** 2021-01-26

**Authors:** Xinyue Li, Guangyu Ji, Juan Zhou, Jingyi Du, Xian Li, Wei Shi, Yong Hu, Wenjuan Zhou, Aijun Hao

**Affiliations:** ^1^Key Laboratory for Experimental Teratology of Ministry of Education, Shandong Key Laboratory of Mental Disorders, Department of Anatomy and Histoembryology, School of Basic Medical Sciences, Cheeloo College of Medicine, Shandong University, Jinan, China; ^2^Department of Foot and Ankle Surgery, Cheeloo College of Medicine, The Second Hospital, Shandong University, Jinan, China; ^3^Department of Blood Transfusion, Qilu Hospital of Shandong University, Jinan, China

**Keywords:** zebrafish, PCGF1, neural induction, neural stem cells, histone methylation

## Abstract

The neural induction constitutes the initial step in the generation of the neural tube. Pcgf1, as one of six Pcgf paralogs, is a maternally expressed gene, but its role and mechanism in early neural induction during neural tube development have not yet been explored. In this study, we found that zebrafish embryos exhibited a small head and reduced or even absence of telencephalon after inhibiting the expression of Pcgf1. Moreover, the neural induction process of zebrafish embryos was abnormally activated, and the subsequent NSC self-renewal was inhibited after injecting the Pcgf1 MO. The results of *in vitro* also showed that knockdown of Pcgf1 increased the expression levels of the neural markers Pax6, Pou3f1, and Zfp521, but decreased the expression levels of the pluripotent markers Oct4, Hes1, and Nanog, which further confirmed that Pcgf1 was indispensable for maintaining the pluripotency of P19 cells. To gain a better understanding of the role of Pcgf1 in early development, we analyzed mRNA profiles from Pcgf1-deficient P19 cells using RNA-seq. We found that the differentially expressed genes were enriched in many functional categories, which related to the development phenotype, and knockdown of Pcgf1 increased the expression of histone demethylases. Finally, our results showed that Pcgf1 loss-of-function decreased the levels of transcriptional repression mark H3K27me3 at the promoters of Ngn1 and Otx2, and the levels of transcriptional activation mark H3K4me3 at the promoters of Pou5f3 and Nanog. Together, our findings reveal that Pcgf1 might function as both a facilitator for pluripotent maintenance and a repressor for neural induction.

## Introduction

Early neural tube development in the embryo includes two important processes: neural induction and self-renewal of neural stem cells (NSCs). Neural induction refers to the process in which the mesoderm induces the ectoderm to develop into a neural plate, and then closes to form a neural tube in the early stage of embryonic development. Later in development, the self-renewal of NSCs is the cytological basis of early neural tube development (Wilson and Edlund, 2001; Hiscock et al., 2018). The neural induction constitutes the initial step in the generation of the vertebrate nervous system. It played a decisive role during early neural tube development (Wilson and Edlund, 2001). The failure of the morphogenetic process of neural tube closure could lead to neural tube defects (Botto et al., 1999; Copp et al., 2013; Greene and Copp, 2014). The abnormal neural tube development causes serious damage to child survival and quality of life (Liao et al., 2016; Blencowe et al., 2018). Therefore, research on the mechanism of neural induction is the key to reveal the pathogenesis of the abnormal neural tube development, which will provide new ideas for its clinical treatment.

The PcG protein family was originally discovered in Drosophila (Lanzuolo and Orlando, 2012) and has always been considered as a class of proteins that inhibit the transcription of target genes at the level of chromatin, which has a stable role in inhibiting transcription through epigenetic modification of histones during stem cell biology and development (Wang et al., 2004; Sparmann and van Lohuizen, 2006; Rajasekhar and Begemann, 2007; Schwartz and Pirrotta, 2008). It includes two protein complexes PRC1 and PRC2 (Di Croce and Helin, 2013). PRC1 complexes can be grouped as canonical PRC1 and non-canonical PRC1 (Simon and Kingston, 2013). The PRC1 complex plays an important role in regulating early embryonic development. It can regulate the differentiation of embryonic stem cells (ESCs) and is closely related to the differentiation process of the three germ layers (Morey et al., 2015; Yang et al., 2016; Endoh et al., 2017; Yan et al., 2017; Zhao et al., 2017; Yao et al., 2018). There is a type of protein in PRC1 that can form a dimer complex with RING1A/B, called Pcgf protein (including six homologous proteins, Pcgf1-Pcgf6) (Di Croce and Helin, 2013). It had been reported that Pcgf2/Mel18 is essential for ESC differentiation into early cardiac-mesoderm precursors (Morey et al., 2015); Pcgf3/5 mainly functions as a transcriptional activator to drive the expression of many genes involved in mesoderm differentiation (Zhao et al., 2017); Pcgf4/Bmi1 is a key regulator of self-renewal of embryonic and adult central nervous system stem cells (Yadirgi et al., 2011); Pcgf6 directly regulates Oct4, Nanog, Sox2, and Lin28 expression to maintain ESC identity (Yang et al., 2016). It is well-documented that the Pcgf protein is implicated in early embryonic development. Here, we focus on Pcgf1, which is mainly expressed in the nervous system (Manoel et al., 2001). *In vitro* studies showed that ESCs deficient in Pcgf1 displayed severe defects in ectoderm and mesoderm differentiation (Yan et al., 2017). Li et al. (2013) reported that Pcgf1 has a positive role in maintaining the pluripotency of P19 cells by directly regulating Oct4. The above studies show that Pcgf1 plays an important role in the early differentiation and pluripotency maintenance, but the biological role of Pcgf1 *in vivo* remains unclear. At present, only one study using the zebrafish model shows that Pcgf1 is involved in early growth, and about 35% of Pcgf1^−/−^ fish show signs of premature aging (Dupret et al., 2016). However, there is still no concern about the role and mechanism of Pcgf1 in early nervous system development.

Early development of zebrafish can be easily observed because of external fertilization and optical transparency of the embryos, which makes it a good model for studying morphogenesis and development of the neural tube (Dahlem et al., 2012; Engert and Wilson, 2012; Kaslin et al., 2013; Schmidt et al., 2013). In our study, we found that the zebrafish embryos exhibited telencephalic malformations after knocking down Pcgf1. In the neural induction stage, the expression of markers related to early neurodevelopment was increased and activated in advance. As a result, the proliferation of NSCs decreased, and cells exited the cell cycle early, which eventually led to abnormal development of the neural tube. P19 cells were derived from an embryo-derived teratocarcinoma, which has the ability to differentiate into each of the three germ layers (Boheler et al., 2002). In the current study, we used P19 cells as a model to investigate the function and mechanism of Pcgf1 in neural induction (Gao et al., 2001; Jin et al., 2009; Xie et al., 2010), which further confirmed the role of Pcgf1 in the neural induction *in vivo*. Thus, our results provide a framework to understand the roles and mechanisms that Pcgf1 participates in the determination of early neurodevelopmental fate.

## Materials and Methods

### Zebrafish Maintenance

Zebrafish maintenance was performed as described previously (Li et al., 2019). Adult zebrafish are reared in a constant-temperature circulating aquaculture system at 28.5°C, and the cycle of 14 h light and 10 h dark is maintained. The fertilized zebrafish embryos were placed in egg water (5 mM NaCl, 0.17 mM KCl, 0.33 mM CaCl_2_, 0.33 mM Mg_2_SO_4_, 10^−5^% Methylene Blue) and were cultured in a constant temperature incubator at 28.5°C. The hatched eggs were fed twice a day, in the morning and evening. Paramecium was used as a first food for zebrafish at 3 dpf when some embryos started to hatch and swim freely to foraging, and then hatched artemia cysts became the only food at 2 weeks post-fertilization. The development of embryo was observed with a stereomicroscope.

### Cell Culture

P19 cells were cultured in α-MEM medium (DMEM; HyClone) that was supplemented with 3% fetal bovine serum (FBS; ExCell Bio), 7% calf bovine serum, 100 U/ml of penicillin, 0.1 mg/ml of streptomycin, and 0.25 μg/ml of amphotericin B. Cells were incubated at 37°C in a humidified atmosphere containing 5% CO_2_.

For RA-induced differentiation, P19 cells were digested with 0.25% trypsin and resuspended with α-MEM medium that was supplemented with 0.5 μM all-trans-RA (Sigma-Aldrich Co. LLC, St. Louis, MO, USA), then replaced with N2 medium after 3–4 days (Chen et al., 2014a).

### Plasmids and Generation of Stably Transfected Cell Lines

Lentivirus-shPcgf1 (U6-MCS-Ubi-EGFP-IRES-Puromycin, shPcgf1 sequence: 5′-GCAGTTTTGACCACTCTAA-3′) was designed by GENECHEM (Shanghai, China). Pcgf1 cDNA was PCR amplified and cloned into the pLVX-EGFP-Puro vector, and lentiviruses were packaged using 293T cells. P19 cells were infected with LV-shPcgf1, LV-Pcgf1, or LV-vector lentivirus for 3 days. The stable cell lines expressing Pcgf1 or Pcgf1-shRNA were selected with puromycin (Chen et al., 2020). The stably infected P19 cells were identified with GFP immunofluorescence and Western blot analysis.

### Bromodeoxyuridine Assay

Zebrafish embryos were collected at stages of 16 and 24 hpf. Embryos were immersed in 10 mM BrdU solution at 28.5°C for 4 h after peeled off the chorion manually with #5 watchmaker's forceps, and then embryos were fixed with 4% paraformaldehyde overnight at 4°C. The next day, embryos were incubated with anti-BrdU antibody overnight and washed with PBST. After incubation with HRP secondary antibody for 1 h, DAB staining was performed to observe BrdU-positive cells (Shi et al., 2016).

### Acridine Orange Staining

Zebrafish embryos were collected at stages of 16 and 24 hpf. Embryos were incubated in 2 μg/ml acridine orange solution at 28.5°C for 30 min in the dark after peeled off the chorion. The embryos were analyzed by fluorescence microscope after washing the embryos with PBS (Shi et al., 2016).

### Microinjection of Morpholino Antisense Oligonucleotides

Morpholine ring-modified antisense oligonucleotides for Pcgf1 was designed in this study to target the translation initiation region of Pcgf1 mRNA. Two Pcgf1-MOs were used in our preliminary experiment, and the one with a more obvious effect was selected in the follow-up experiment. The sequence is 5′-CCTTGCTCCGCCATCTTTGGGAATT-3′. The standard control MO (5′-CCTTcCTCCcCCATgTTaGcGAATT-3′) was five mismatches with regard to Pcgf1 MO. The defects in morphogenic events were dose dependent. In the following study, we chose 5 ng/embryo as the experimental concentration, which resulted in the highest proportion of mild phenotype and the lowest death rate (Figure S1). Human Pcgf1 mRNA was transcribed from linearized pCS2+ constructs *in vitro* using SP6 RNA polymerase (Thermo Fisher). Synthetic mRNA was injected into a one-cell stage combined with MO for rescue experiments (Chen et al., 2014c).

### RNA Extraction and Quantitative Real-Time PCR Assays

Total RNA was extracted from different stages of embryos or P19 cells following the standard protocols using TRIzol reagent (TRANS). Thirty embryos of every stage or about 1–5 × 10^7^ P19 cells are needed (Chen et al., 2014b; Yin and Hu, 2014). Total RNA was extracted, and then the concentration was measured by a spectrophotometer. Total RNA (1 μg) was converted to cDNA by using the RevertAid^TM^ First Strand cDNA Synthesis Kit (Thermo Fisher Scientific). The reverse transcription system is as follows: 1 μg of total RNA, 1 μl of oligo (dT) primer (100 μM), and nuclease-free water (with total volume of 12 μl) were mixed and incubated at 65°C for 5 min. Then the following components were added and incubated for 60 min at 42°C: 4 μl of 5× reaction buffer, 1 μl of RiboLock RNase inhibitor (20 U/μl), 2 μl of 10 mM dNTP mix, and 1 μl of RevertAid RT (200 U/μl). This was followed by heating at 70°C for 5 min to terminate the reaction. Real-time PCR was performed with SYBR Green Realtime PCR Master Mix (TOYOBO CO., Ltd., Japan) according to the manufacturer's instructions. The qPCR reaction system is as follows: 10 μl of SYBR Green Realtime PCR Master Mix, 0.8 μl of PCR forward primer (10 μM), 0.8 μl of PCR reverse primer (10 μM), 2 μl of template cDNA, and 6.4 μl of ddH_2_O. The qPCR reaction conditions were set as follows: initial denaturation at 95°C for 5 min, 40 PCR cycles including denaturation at 95°C for 10 s, annealing at 60°C for 20 s, and extending at 72°C for 30 s. Melting curve analysis was performed at the end of each reaction. The expression of β-actin gene represented internal controls, and the relative expression of genes was calculated with the 2^−ΔΔCT^ method. The primer sequences were as follows: zebrafish: actin (forward, 5′-GCTGCCTCTTCTTCCTCC-3′; reverse, 5′- ATGTCCACGTCGCACTTC-3′), Pcgf1 (forward, 5′-GAAGTACGGCATTTGAGG-3′; reverse, 5′-CTATCTCGTCCGCTTGTC-3′), Sox2 (forward, 5′-GAACCCCAAAATGCACAATTCG-3′; reverse, 5′-ACTTGTCCTTCTTCATCAGGGT-3), Sox3 (forward, 5′-CCATTCCGCAGTCCAACA-3′; reverse, 5′-GATTCTCCTGAGCCATCTTC-3′), Otx2 (forward, 5′-ATGTCGTATCTCAAGCAACCAC-3′; reverse, 5′-GTCCTTTCTCGTCTCTGCTTTC-3′), Ngn1 (forward, 5′-CGTCGTGAAGAAGAACCG-3′; reverse, 5′-CTCCGAAAGTGCCCAGAT-3′), P21 (forward, 5′-TGTCAGGAAAAGCAGCAGAAAC-3′; reverse, 5′-CGCTTCTTGGCTTGGTAGAAAT-3′), P57 (forward, 5′-TAAACTCCAAACCAGCTCGTTC-3′; reverse, 5′-CGTTACTTCAATGCTCGTGGAT-3′), Smad1 (forward, 5′-GCTAAACTCTCCATGCTGCCC-3′; reverse, 5′-GCGAGCTGGGATAACTGTTG-3′), Smad4 (forward, 5′-GAGCAGGAACAGTAACTTCACC-3′; reverse, 5′-GTCCATCTCGAAGTAGGCAATG-3′), Smad5 (forward, 5′-TGAGTCACAACGAGCCTCAT-3′; reverse, 5′-CTTGCAGGAGAGTTGGGGTA-3′), Wnt3a (forward, 5′-TCACTGACCACATGTACCTGAA-3′; reverse, 5′-TTCTCAACCACCATTTCCGATG-3′), Wnt8a (forward, 5′-TTTTGCGTCGTTGGTTATGTCT-3′; reverse, 5′-CTGCTGGTGTATGCGAGATAAG-3′), β-catenin (forward, 5′-GCAGATACCTTCCACACAGTTC-3′; reverse, 5′-CTGCCTTATTAACCACCACCTG-3′). Mouse: Actin (forward, 5′-CGTTGACATCCGTAAAGACCTC-3′; reverse, 5′-CCACCGATCCACACAGAGTAC-3′), Pcgf1 (forward, 5′-TGAAGTACCTGCAAACCA-3′; reverse, 5′-AAGCCTCGGGACTGATAG-3′), Pou3f1 (forward, 5′-TCGAGGTGGGTGTCAAAGG-3′; reverse, 5′-GGCGCATAAACGTCGTCCA-3′), Nanog (forward, 5′-TTGCTTACAAGGGTCTGCTACT-3′; reverse, 5′-ACTGGTAGAAGAATCAGGGCT-3′), Zpf521 (forward, 5′-CCTGACTGGGTTTCGTT-3′; reverse, 5′-CTCTTTGAGGCAAGATGC-3′), Oct4 (forward, 5′-GAAGAGTATGAGGCTACAGGG-3′; reverse, 5′-AGCAGTGACGGGAACAGA-3′), Hes1 (forward, 5′-CGAGCGTGTTGGGGAAGTA-3′; reverse, 5′-AGTGCGCACCTCGGTGTTA-3′), Pax6 (forward, 5′-TGGGAAATCCGAGACAGA-3′; reverse, 5′-GCCCGTTCAACATCCTTA-3′).

### Whole-Mount *in situ* Hybridization

The templates of antisense RNA probes for WISH were amplified by PCR with the primer specific for each gene listed above. PCR products were then cloned into the pEASY-T3 vector (TRANS). Then the pEASY-T3-derived plasmids were linearized. Antisense RNA probes were transcribed from linearized templates by T7 or SP6 polymerases (Thermo) in the presence of DIG-labeled nucleotides (Roche, Mannheim, Germany), and *in situ* hybridization was carried out as described below using the antisense RNA probes prepared (Chen et al., 2013).

Zebrafish embryos were fixed with 4% paraformaldehyde in PBS at 4°C overnight at different developmental stages (eight cells, sphere, shield, 75% epiboly, 10, 16, 24 hpf). The fixed embryos were pretreated with proteinase K (10 μg/ml) for 10 min, washed by PBST, and then the egg chorion was removed. The embryos were subjected to prehybridization in Hyb– (50% deionizing formamide buffer + 25% 20x SSC + 0.1% Tween) for 5 min and Hyb+ (98.9% Hyb– + 1% 5 mg/ml Yeast RNA + 0.1% 50 mg/ml Heparin sodium salt) for 4 h at 65°C, then they were incubated in 1 ng/μl antisense RNA probes for hybridization overnight at 65°C. The next day, the embryos were washed with Liquid I (50% deionizing formamide buffer + 10% 20x SSC + 0.1% Tween), Liquid II (10% 20x SSC + 0.1% Tween), Liquid III (10% Liquid II + 0.1% Tween), and blocked in blocking solution [1% blocking powder (Roche, 11096176001) and 10% goat serum (ORIGENE, ZLI-9021) dissolved in Maleic Acid Buffer] to block the non-specific binding sites at room temperature for 4 h. The embryos were then incubated with anti-DIG-AP (Roche, 11093274910, 1:2,500) at 4°C overnight. On the third day, NBT/BCIP (Roche, 11681451001, 1:50) was used for color detection, and images were acquired with a stereomicroscope (Olympus, SZX16) (Thisse and Thisse, 2008).

### Western Blotting

Total protein of P19 cells (1–5 × 10^7^) and zebrafish embryos (25–30 embryos) were lysed in RIPA buffer with protease inhibitors on ice. The supernatants were collected by centrifuging at 12,000 rpm at 4°C for 15 min. The supernatants were then quantified using the bicinchonininic acid (BCA) assay. The samples were mixed with loading buffer, and equal amounts of proteins (20–30 μg) were separated by SDS-polyacrylamide gel and transferred to PVDF membranes. The membranes were incubated with 5% skimmed milk for 2 h and probed overnight at 4°C with the indicated primary antibodies. The primary antibodies used were as follows: Pcgf1 (ab84108, 1:1,000), Pax6 (ab195045, 1:1,000), Oct4 (ab18976, 1:1,000), H3K4me3 (ab8580, 1:1,000), and H3K27me3 (ab6002, 1:1,000) were from Abcam; GFP (#2956, 1:1,000) and H3 (#4499, 1:1,000) were from Cell Signaling Technology, and β-actin (HC201-01, 1:1,000) was from TransGen Biotech. Then secondary antibodies were incubated for 1 h at room temperature after washing. Secondary antibodies were HRP conjugated to either goat anti-mouse or anti-rabbit IgG antibodies. Protein bands were visualized by Immobilon^™^ Western Chemiluminescent HRP Substrate (MLLIPORE), and immunoreactive protein bands were analyzed by densitometry using Quantity one.

### RNA Sequencing

To perform RNA sequencing (RNA-seq), wild-type P19 cells and Pcgf1-KD P19 cells were harvested and lysed by TRIzol, and total RNA was collected according to the manufacturer's instructions. We mixed RNA samples from three independent experiments, and RNA-seq was carried out on a BGISEQ-500 (Beijing Genomic Institution, www.genomics.org.cn, BGI). The significance of the differential expression of genes was confirmed by the bioinformatics service of BGI to the combination of absolute value of log2-ratio ≥ 1 and FDR ≤ 0.001. The original sequence data were submitted to the database of NCBI Sequence Read Archive.

### Chromatin Immunoprecipitation Assay

The ChIP assay was performed as reported earlier (Li et al., 2019). Briefly, protein–DNA complexes of zebrafish embryos were cross-linked by treatment with 1% formaldehyde for 15 min, and then glycine was used to stop cross-linking. Chromatin was sonicated to shear into 20–500 bp. Equal aliquots of isolated chromatin were immunoprecipitated with H3K27me3 and H3K4me3 antibody or a control antibody (anti-IgG) overnight at 4°C with rotation, followed by incubation with protein G agarose for 1 h. The DNA fragments associated with specific immunoprecipitates were purified and used as templates for real-time PCR. The ChIP qPCR primer sequences were as follows: Ngn1 (forward, 5′-ACTTGATGCCAGCGAAAG-3′; reverse, 5′-CTGCGACACTCCAATAGC-3′), Otx2 (forward, 5′-TATGTTGCTCACCGTAGT-3′; reverse, 5′-GAGGAAAGCCGACTCTAT-3′), Pou5f3 (forward, 5′-AGGTGGGAGATGTGACGC-3′; reverse, 5′-GACCAGGAGTGACAAATA-3′), and Nanog (forward, 5′-CTTCAATCAGCATCCGTTTTC-3′; reverse, 5′-ACTCAAGCAGAAAGTAACGT -3′).

### Statistical Analysis

Data are presented as mean ± SEM of at least three independent experiments. The statistical analyses were performed with the Student's *t-*test for comparison of the two groups. One-way ANOVA was performed for three or more groups. *P* < 0.05 was considered as statistically significant.

## Results

### Expression Pattern of Pcgf1 During Zebrafish Embryonic Development

**Figure 1:**
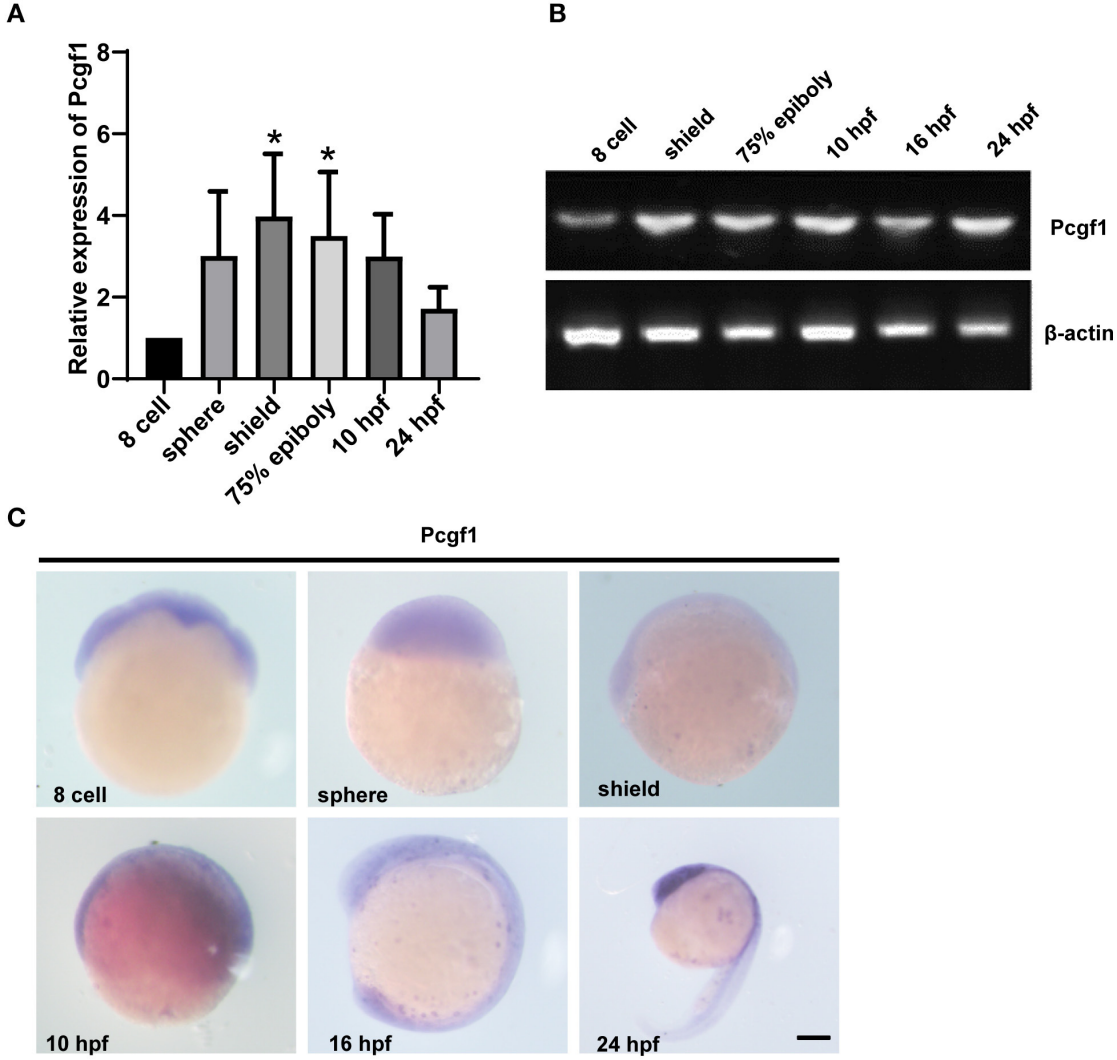
The expression pattern of Pcgf1 during zebrafish embryonic development. **(A,B)** qPCR and RT-PCR were used to analyze the expression level of the Pcgf1 during zebrafish developmental stages, ranging from eight cells to 24 hpf. Pcgf1 is maternally expressed, increased at sphere phase, and continued to 24 hpf ^*^P < 0.05. **(C)** The expression of Pcgf1 in zebrafish was determined by whole-mount *in situ* hybridization at the indicated stages (from eight cells to 24 hpf). Scale bar: 200 μm.

We first analyzed the spatiotemporal expression characteristics of the Pcgf1 at different stages of embryonic development by RT-PCR, qPCR, and *in situ* hybridization. Our results showed that the mRNA expression of Pcgf1 at different stages was as follows: Pcgf1 is maternally expressed during zebrafish embryonic development. The expression of Pcgf1 increased significantly at shield phase. At shield stage, embryonic development enters the gastrointestinal embryo stage and begins to differentiate into the three germ layers, which is a key period of neural induction. It is suggested that Pcgf1 is specific in the early stage of neural development, especially in the period of neural induction (Figures 1A,B). The results of *in situ* hybridization showed that Pcgf1 had a certain specificity at 16 hpf, mainly concentrated in the central nervous system and the entire neural tube region, especially at the head and tail. At 24 hpf, Pcgf1 was abundant in the forebrain (Figure 1C).

### Knockdown of Pcgf1 Causes Deficits in the Development of Neural Tubes

**Figure 2:**
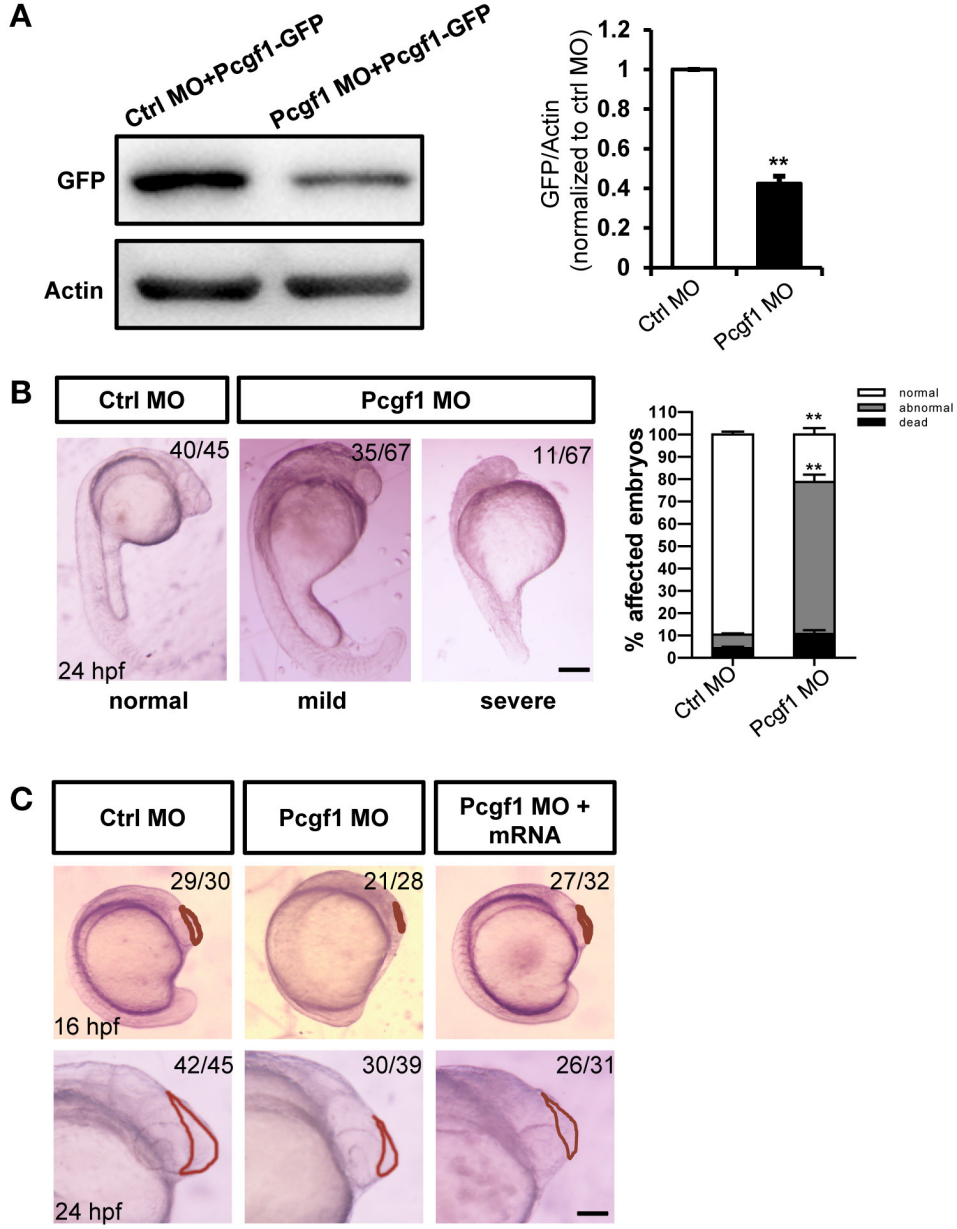
Zebrafish embryos displayed telencephalic malformations after knockdown of Pcgf1. **(A)** Western blot analysis of the specificity and efficiency of Pcgf1 knockdown in zebrafish at 24 hpf. The Pcgf1 MO could efficiently knockdown the expression of Pcgf1 because the expression of GFP is downregulated. The bar graphs clarified the relative level of GFP/actin expression for at least three independent experiments. Values represent mean ± SEM. ^**^P < 0.01. **(B)** Compared with the embryos in the control group, the development of neural tube in the experimental group (MO) had obvious defects. The mild phenotype consisted of a smaller head and a shorter body axis, and the severe phenotype had a partial head area deletion. Bar graphs show the statistical data for the embryo numbers. ^**^P < 0.01. **(C)** Morphology of Pcgf1 morphants at 16 and 24 hpf. The Pcgf1 MO group showed that the area of the telencephalon decreased or even disappeared compared with the control group. Co-injections of human Pcgf1 mRNA with Pcgf1 MO could partially rescue the telencephalic defects induced by Pcgf1 MO. Red line point to the telencephalic region. Scale bar: 200 μm.

To verify the role of Pcgf1 during zebrafish embryonic development, we knocked down Pcgf1 in zebrafish embryos using morpholino (MO) antisense oligonucleotides. The efficiency of MO treatment was assessed by Western blot after coinjection with Pcgf1 MO or control MO with GFP-tagged Pcgf1 mRNA. The results confirmed that the MO oligonucleotides inhibit the translation of Pcgf1 mRNA (Figure 2A). The phenotype of zebrafish embryos showed that knockdown of Pcgf1 caused malformation in telencephalon at 16 and 24 hpf with a small head, reduced or even absence of telencephalon, and shortening of body axis (Figures 2B,C). In order to assess whether the telencephalic malformations were specific to the lack of Pcgf1, we used rescue experiments by co-injections of Pcgf1 mRNA with Pcgf1 MO. Our results showed that the expression of Pcgf1 could partially rescue the MO-induced telencephalon defects (Figure 2C).

### Knockdown of Pcgf1 Leads to Abnormal Activation of Neural Induction

**Figure 3:**
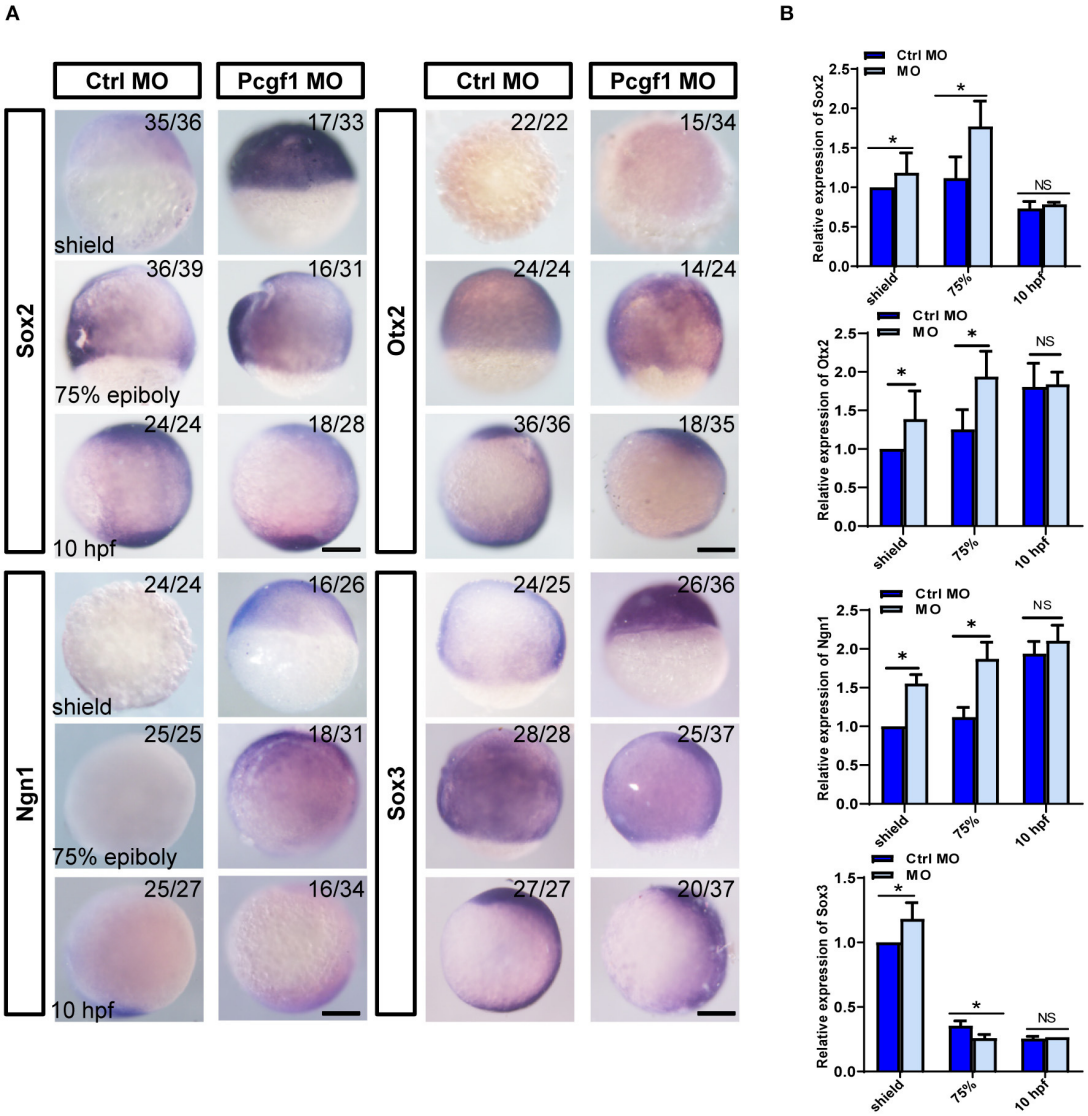
Effect of Pcgf1 on neural induction phase. **(A)** The expression levels of Sox2, Otx2, Ngn1, and Sox3 were detected by whole-mount *in situ* hybridization at neural induction phase (shield phase, 75% epiboly phase, and 10 hpf). Scale bar: 200 μm. **(B)** The expression of Sox2, Otx2, Ngn1, and Sox3 was analyzed by qPCR, and the expression of β-actin gene represented internal controls. Data represent the mean of at least three independent experiments ± SEM, ^*^P < 0.05 vs. control MO.

To further investigate the relationship between telencephalon deletion caused by Pcgf1 knockdown and the process of neural induction, we analyzed the expression of several neural markers during neural induction phase (shield, 75% epiboly phase, and 10 hpf) of zebrafish embryonic development. The results of *in situ* hybridization showed that the expression of neural markers Sox2, Otx2, and Ngn1 was activated abnormally compared with the control group, and the expression of Sox3 increased at shield phase and decreased from 75% epiboly phase (Figure 3A). In order to further verify the above conclusions, we tested the expression of neural markers by qPCR, and the results were consistent with those of *in situ* hybridization (Figure 3B). These results suggested that knockdown of Pcgf1 led to abnormal activation of neural induction.

### Knockdown of Pcgf1 Inhibits the Proliferation of Neural Stem Cells

**Figure 4:**
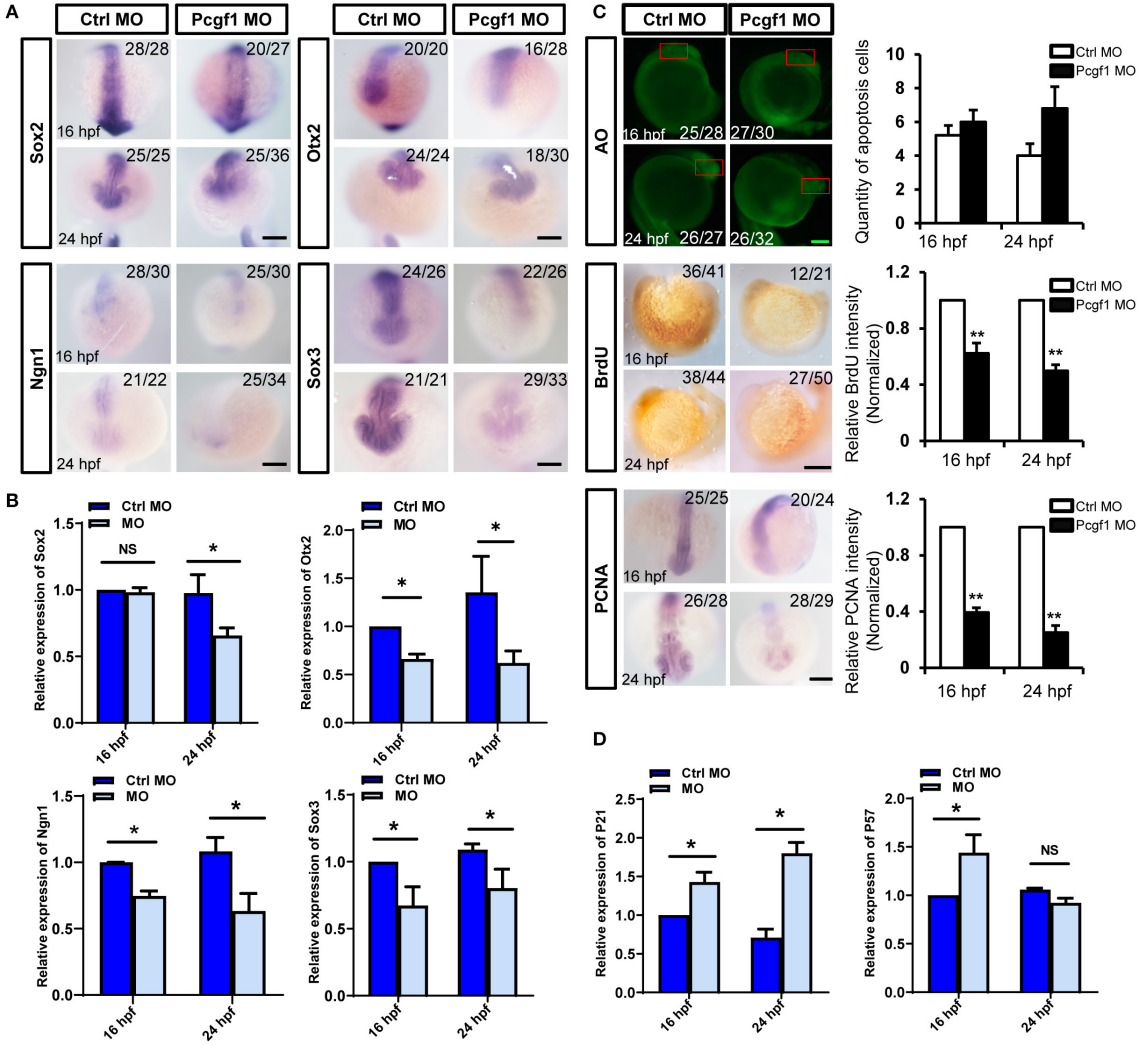
Effect of Pcgf1 on neural stem cells (NSCs) in neural tubes. **(A)** The expression levels of Sox2, Otx2, Ngn1, and Sox3 were detected by whole-mount *in situ* hybridization at self-renewal phase of NSCs (16 and 24 hpf). Scale bar: 200 μm. **(B)** The expression of Sox2, Otx2, Ngn1, and Sox3 was analyzed by qPCR, and the expression of β-actin gene represented internal controls. Data represent the mean of at least three independent experiments ± SEM, ^*^P < 0.05 vs. control MO. **(C)** Acridine orange staining was used to detect apoptosis at 16 and 24 hpf; BrdU labeling of control MO and Pcgf1 MO-injected embryos at 16 and 24 hpf. The expression of PCNA was detected by whole-mount *in situ* hybridization. Data represent the mean of at least three independent experiments ± SEM, ^**^P < 0.01 vs. control MO. **(D)** The expression of p21 and p57 were analyzed by qPCR, and the expression of β-actin gene represented internal controls. Data represent the mean of at least three independent experiments ± SEM. ^*^P < 0.05 vs. control MO.

The proliferation, apoptosis, differentiation, and migration of NSCs are also the cytological basis for the normal development of neural tubes. It is not clear whether the early activation of neural induction phase caused by Pcgf1 knockdown could affect the subsequent self-renewal of NSCs. We first analyzed the expression of several neural markers during NSC self-renewal phase (16 and 24 hpf) of zebrafish embryonic development. The results of *in situ* hybridization and qPCR showed that the expression of all the neural markers Sox2, Otx2, Ngn1, and Sox3 were decreased significantly during NSC self-renewal phase (Figures 4A,B). Moreover, BrdU incorporation assay and PCNA (a well-accepted marker of proliferation) *in situ* hybridization were used to detect the effect of Pcgf1 on the proliferation of NSCs. The results showed that the level of BrdU incorporation and the expression level of PCNA were decreased in the Pcgf1 MO group. At the same time, acridine orange (AO) staining was used to detect the effect of Pcgf1 on the apoptosis of NSCs. The results showed that there was no significant change in apoptosis (Figure 4C). Cyclin-dependent kinase (CDK) inhibitors are important for neural differentiation. We found that, compared with the control group, the expression of the CDK inhibitors p21 and p57 was increased after injecting Pcgf1 MO (Figure 4D). Taken together, these results suggested that the abnormal development of neural tube might be caused by the abnormality in the neural induction phase and the subsequent impaired proliferation and premature differentiation of NSCs after injecting the Pcgf1 MO.

### Pcgf1 Is Indispensable for Maintaining the Pluripotency of P19 Cells

**Figure 5:**
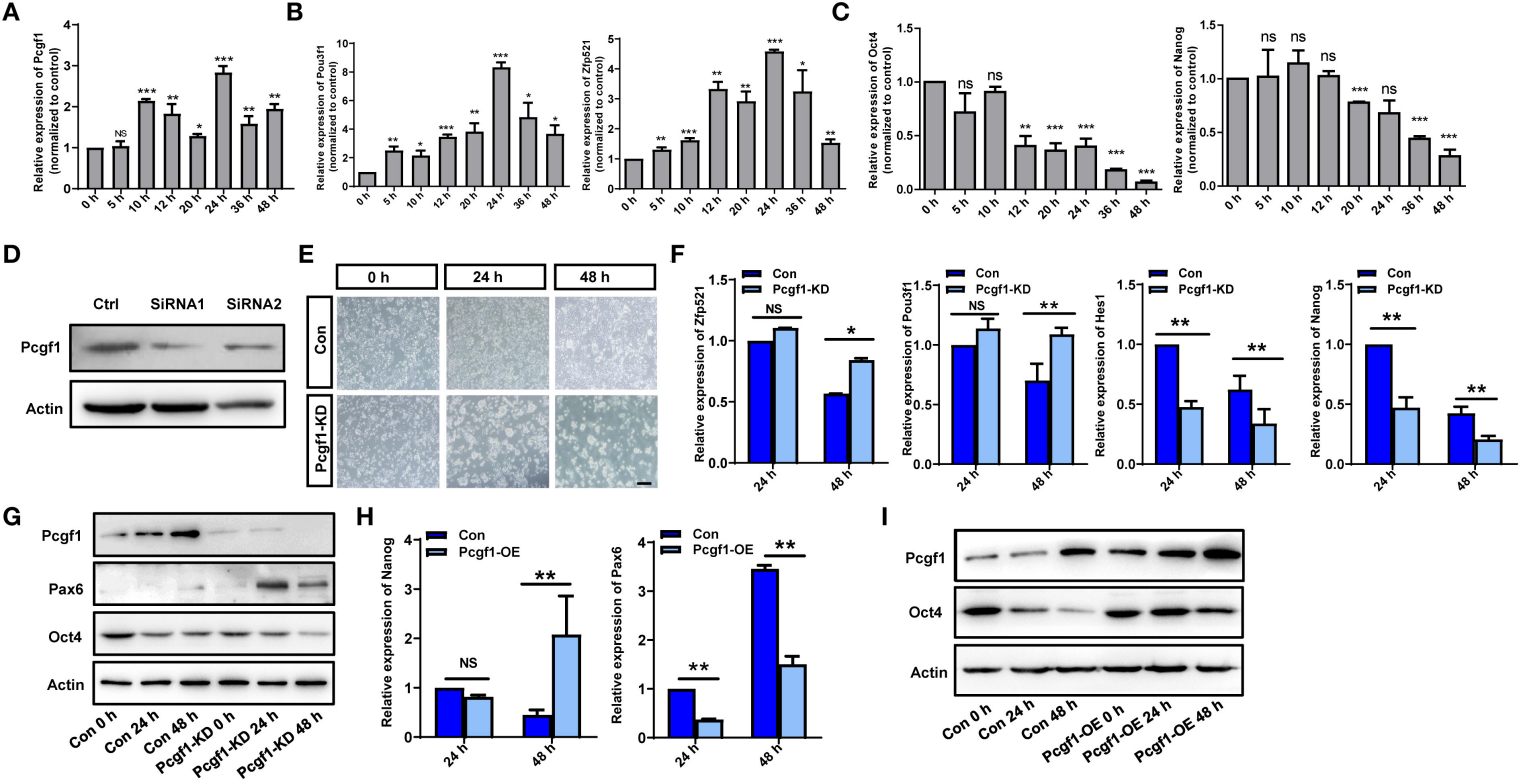
Pcgf1 had a positive role in maintaining the pluripotency of P19 cells. **(A)** qPCR showed that the expression of Pcgf1 was increased in P19 cells induced by retinoic acid, especially 24 h later. **(B,C)** The expression of neural markers Pou3f1 and Zfp521 were increased, while the pluripotent markers Oct4 and Nanog were decreased. **(D)** Construction of stable P19 cell line with Pcgf1 knockdown by lentivirus. **(E)** P19 cells clustered earlier than the control group after Pcgf1 knocked down at 24 and 48 h induced by RA. Scale bar, 100 μm. **(F)** The expression levels of the neural markers Pou3f1 and Zfp521, and the pluripotent markers Hes1 and Nanog, were detected by qPCR after Pcgf1 knock down at 2 and 48 h induced by RA. **(G)** The expression levels of the neural marker Pax6 and the pluripotent marker Oct4 were detected by Western blot after Pcgf1 knock down at 0, 24, and 48 h induced by RA. **(H)** The expression levels of the neural marker Pax6 and the pluripotent marker Nanog were detected by qPCR after overexpressed Pcgf1 at 24 and 48 h was induced by RA. **(I)** The Western blot results showed that pluripotent marker Oct4 was consistently expressed after overexpressed Pcgf1 at 0, 24, and 48 h was induced by RA. Data represent the mean of at least three independent experiments ± SEM. The expression of β-actin gene represented internal controls. ^*^P < 0.05 vs. control, ^**^P < 0.01 vs. control, ^***^P < 0.001 vs. control.

P19 cells stimulated by retinoic acid (RA) can differentiate into neural ectoderm and endoderm-derived cells, which was used to study the process of neural induction (Gao et al., 2001; Xie et al., 2010). First, we found that the expression of Pcgf1 was increased with the increase in the neural markers Pou3f1 and Zfp521 and the decrease in the pluripotent markers Oct4 and Nanog in RA-induced P19 cells (Figures 5A–C). In order to explore the effect of Pcgf1 on cell pluripotency, we constructed a stable P19 cell line that knocked down Pcgf1 (Pcgf1-KD) or overexpressed Pcgf1 (Pcgf1-OE) and treated it with RA. We observed the morphological changes of P19 cells induced by RA after Pcgf1 knocked down. Compared with the control group, P19 cells aggregated earlier at 24 h after Pcgf1 knocked down, indicating that P19 cells entered the neural induction stage in advance (Figures 5D,E). Then we examined the effects of Pcgf1 on the mRNA and protein levels of cell pluripotent genes. QPCR and Western blot analysis showed that the expression of the neural markers Pax6, Pou3f1, and Zfp521 increased, while the pluripotent markers Oct4, Hes1, and Nanog decreased after knocking down of Pcgf1 (Figures 5F,G). Overexpression of Pcgf1 resulted in a decrease in the neural marker Pax6 at 24 and 48 h, and the increase in pluripotent marker Nanog at 48 h in RA-induced P19 cells (Figure 5H). Western blot showed that overexpression of Pcgf1 could inhibit the decrease of Oct4 induced by RA (Figure 5I). It is suggested that Pcgf1 may maintain the pluripotency of P19 cells.

### Pcgf1 Regulates Neural Induction Through an Epigenetic Mechanism

**Figure 6:**
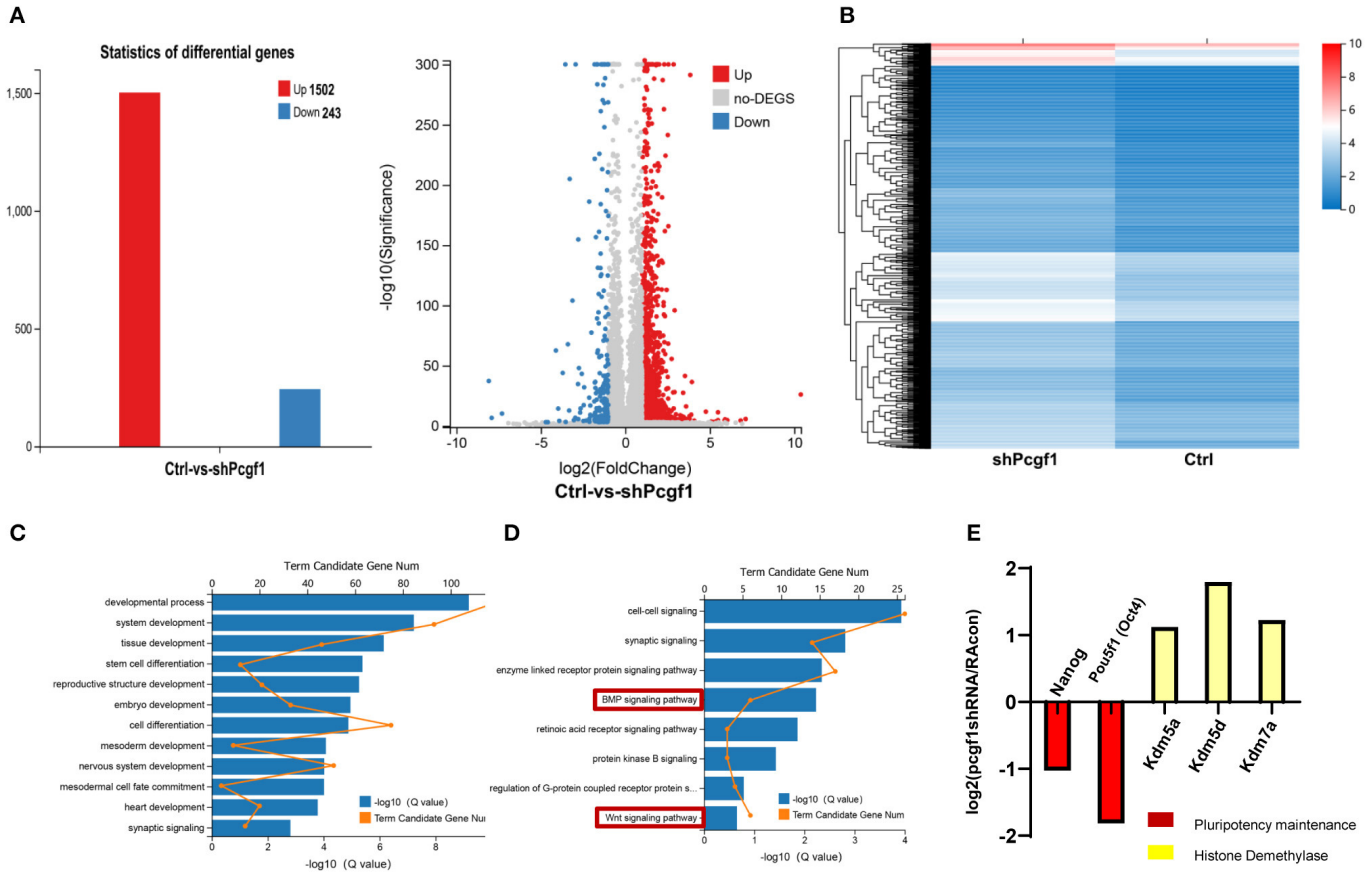
Pcgf1 regulated neural induction through an epigenetic mechanism in addition to signaling pathways. **(A)** Histogram and volcano plots represented differentially expressed genes in wild type and Pcgf1-KD P19 cells. The red color indicates upregulated genes, and the blue color indicates downregulated genes if they had a log2 fold change of >1 or < −1, respectively. The differentially expressed gene number was indicated at right. **(B)** A heat map of genes with more than 2-fold expression differences in wild-type and Pcgf1-KD P19 cells. Red indicates a high expression, and blue indicates a low expression. **(C)** Gene ontology (GO) analysis of biological functions of deregulated genes in Pcgf1-KD P19 cells. **(D)** GO analysis of signaling pathways of deregulated genes in Pcgf1-KD P19 cells. **(E)** Fold changes of the expression levels of pluripotent markers (nanog and pou5f1) and histone demethylase (kdm5a, kdm5d, and kdm7a) in RNA-seq results.

To understand the underlying mechanism by which Pcgf1 affects neural induction and monitors the dynamic changes in gene expression during neural induction phase upon Pcgf1 loss-of-function, we performed RNA-seq analysis on wild-type and Pcgf1-KD P19 cells. RNA-seq analysis identified 1,745 genes with more than 2-fold altered expression levels, with 1,502 genes upregulated, while only 243 genes were downregulated in the absence of Pcgf1 (Figure 6A). Heat map revealed the genes with more than 2-fold expression differences in wild-type and Pcgf1-KD P19 cells (Figure 6B). Next, we used gene ontology (GO) analysis to identify the functions of the significantly downregulated genes. These genes were enriched in many functional categories, which conformed to the development phenotype we observed, like the development of mesoderm, embryo, and nervous system (Figure 6C). GO analysis also revealed that the downregulated genes were mainly involved in the neural induction phase-related signaling pathways, like the BMP and Wnt signaling pathways (Figure 6D). Figure 6E showed that markers related to the maintenance of pluripotency (Nanog and Pou5f1) were significantly downregulated, suggesting that the pluripotency is reduced and cells entered the neural induction stage in advance after Pcgf1 knocked down. Meanwhile, the expression levels of histone demethylases (Kdm5a, Kdm5d, and Kdm7a) were upregulated significantly, which indicated that Pcgf1 may regulate neural induction through an epigenetic mechanism in addition to regulating the signaling pathways.

### Knockdown of Pcgf1 Affects H3K27me3 and H3K4me3 Distributions During Neural Induction

**Figure 7:**
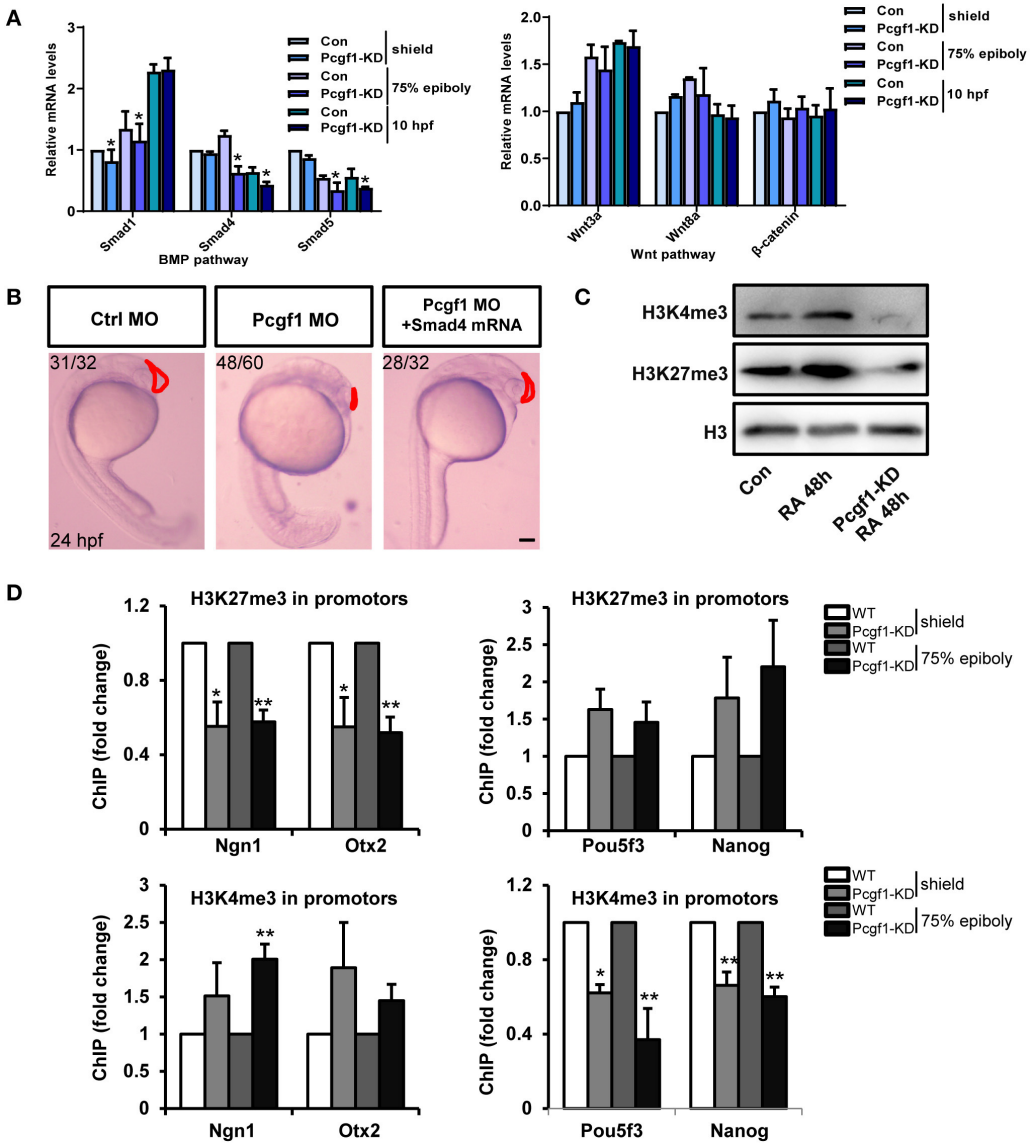
Pcgf1 regulated neural induction through histone methylation. **(A)** qPCR analysis of the expression levels of BMP signaling pathway (Smad1, Smad4, and Smad5) and Wnt signaling pathway (wnt3a, wnt8a, and β-catenin). Compared with the controls, the mRNA levels of Smad1, Smad4, and Smad5 in Pcgf1 MO-injected embryos decreased, but the levels of wnt3a, wnt8a, and β-catenin did not change observably. Data represent the mean of at least three independent experiments ± SD. ^*^P < 0.05 vs. control. **(B)** Compared with the embryos injected with Pcgf1 MO, the shrinking of the telencephalon has not been rescued by the addition of Smad4 mRNA. **(C)** Western blot showed that the expression of H3K4me3 and H3K27me3 decreased after Pcgf1 knocked down. **(D)** The WT and Pcgf1 MO-injected group were immunoprecipitated with anti-H3K27me3, anti-H3K4me3, and IgG. The isolated DNA was analyzed by gene-specific ChIP primers. The levels of H3K27me3 at the promoters of Ngn1 and Otx2, and the levels of H3K4me3 at the promoters of Pou5f3 and Nanog, were significantly decreased after injection with the Pcgf1 MO. Data represent the mean of at least three independent experiments ± SD. ^*^P < 0.05, ^**^P < 0.01.

In zebrafish, the regulation of the neural induction process involves the coordination of multiple signaling pathways, such as Wnt and BMP. GO analysis showed that these two signaling pathways could be affected by Pcgf1. Our results showed that the activity of the BMP signaling pathway (Smad1, Smad4, and Smad5) was decreased, especially Smad4, but the activity of key molecules (Wnt3a, Wnt8a, and β-catenin) in the Wnt signaling pathway did not change observably after knocking down Pcgf1 (Figure 7A). However, injection with Smad4 mRNA could not rescue the most obvious phenotype caused by Pcgf1 knockdown, such as telencephalic loss (Figure 7B). These results suggest that there may be another mechanism by which Pcgf1 regulates the neural induction process.

PcG genes are identified as essential factors in epigenetic developmental processes. Epigenetic modification of chromatin structure results in the activation or silencing of specific genes, which has been proven to be an important molecular mechanism in development and disease. In our study, we found that the expression levels of H3K4me3 and H3K27me3 were both decreased after Pcgf1 knockdown (Figure 7C). ChIP-qPCR results further showed that the levels of H3K27me3 at the promoters of Ngn1 and Otx2, and the levels of H3K4me3 at the promoters of Pou5f3 and Nanog, were significantly decreased after injection of Pcgf1 MO (Figure 7D). We also found that there was no decrease in the levels of H3K27me3 at the promoters of Pou5f3 and Nanog, and the levels of H3K4me3 at the promoters of Ngn1 and Otx2, which suggested that the decrease in binding level of the above gene is specific. Overall, the above results suggested that Pcgf1 may be involved in the regulation of neural development through histone modification, including transcriptional repression and transcriptional activation mechanisms: Pcgf1 may inhibit the expression of genes related to neurodevelopment through H3K27me3 and promote the expression of pluripotent genes through H3K4me3.

## Discussion

**Figure 8:**
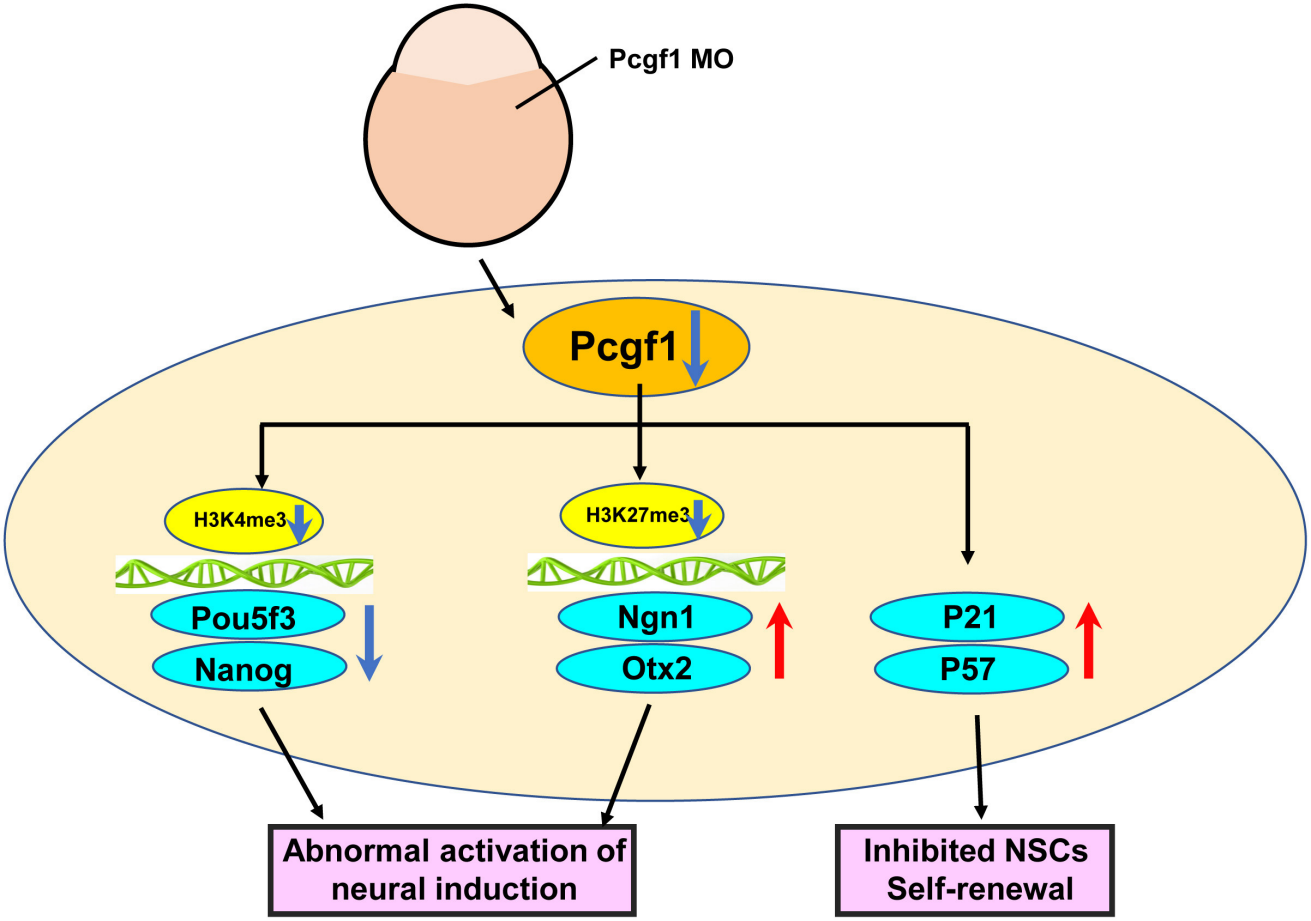
Schematic representation of the effect of Pcgf1 on the neural induction and NSC self-renewal.

Neural induction is defined as the process of specification of ectodermal cells into neural stem or precursor cells, which play essential roles in early neural tube development. To study the molecular regulation of neural induction is the key to reveal the pathogenesis of the abnormal neural tube development. Here, we showed that Pcgf1 is maternally expressed during the embryonic development of zebrafish. However, the biological function of Pcgf1 in the early neural tube development is still unclear. In order to study the effect of Pcgf1 on the early neural tube development, we constructed Pcgf1 knockdown zebrafish as an animal model. We found that loss of Pcgf1 resulted in telencephalon malformations (small head, reduced telencephalon, andshortened body axis) in zebrafish embryos. Furthermore, knock down of Pcgf1 resulted in the abnormal neural induction process, which led to the decrease in NSC proliferation in the zebrafish embryo. Finally, our results showed that Pcgf1 regulated the levels of H3K27me3 in the Ngn1 and Otx2 promoter regions, and the levels of H3K4me3 at the promoters of Pou5f3 and Nanog, which explained the role of Pcgf1 in neural induction from the perspective of epigenetics (Figure 8).

The PRC1 complex functions as a transcriptional repressor in stem cell biology and development. They are divided into six groups based on the critical factors Pcgf genes. Although it had been reported that Pcgf1-6 play important roles in the self-renewal, proliferation, and differentiation of ESC (Yadirgi et al., 2011; Morey et al., 2015; Yang et al., 2016; Zhao et al., 2017), and the expression levels of Pcgf family members at different stages of early embryonic development have been detected (Manoel et al., 2001; Dupret et al., 2016; Chrispijn et al., 2018), the biological functions of each Pcgf family member, especially in the early neural tube development, are still unclear. Here, we first analyzed the expression of Pcgf1 at different stages of early zebrafish embryonic development. Our results further confirmed the expression characteristics that Pcgf1 is maternally expressed and highly expressed from sphere to 10 hpf of zebrafish embryonic development, which was consistent with the previous studies (Dupret et al., 2016). Meanwhile, the expression region of Pcgf1 in zebrafish embryos was mainly concentrated in the nervous system at 24 hpf, which was consistent with the high expression of Pcgf1 in the nervous system of mouse embryos, especially in the neural tube (Gong et al., 2005). However, the biological function of Pcgf1 *in vivo* is still unclear. To investigate the function of Pcgf1, we inhibited the expression of Pcgf1 in zebrafish embryos using MO oligonucleotides. We found that after the injection of Pcgf1 MO, embryos exhibited obvious morphogenesis defects with a small head, reduced or even disappeared telencephalon, and shortening of the body axis by 24 hpf. In fact, a previous study has reported that Pcgf1 knockout leads to retarded growth and abnormal craniofacial development in zebrafish, which proves that Pcgf1 plays an important role in the early developmental stages and also supports our conclusion to a certain extent (Dupret et al., 2016). However, it did not pay much attention to the role and mechanism of Pcgf1 in early telencephalon development. Therefore, this is the first time that the role of Pcgf1 was found in early neurodevelopment *in vivo*, which will be a breakthrough point to study the role of Pcgf1 in early neurodevelopment. In addition, disruption of Ring 1b/Rnf2, the E3 ubiquitin ligase in PRC1 in mice, leads to an arrest at gastrulation, and Ring 1b mutants in zebrafish displayed several defects, including jaw malformations, and they died at around 4–5 dpf (Voncken et al., 2003; van der Velden et al., 2012). Ring 1b/Rnf2 is also required for craniofacial development, and zebrafish Ring 1b mutants display a severe craniofacial phenotype (van der Velden et al., 2013; Chrispijn et al., 2019). This may be due to the important role of Pcgf1 in the development of embryonic stem cells.

The neural induction process and NSC self-renewal are two most important processes of neural tube development. However, most of the studies on Pcgf1 are carried out *in vitro*, and the role of Pcgf1 in neural development *in vivo* has not been fully revealed. In the neural differentiation process of RA-induced P19 cells, Pcgf1 can positively regulate the Oct4–Nanog–Sox2 axis, thus maintaining the pluripotency of P19 cells (Li et al., 2013). In embryonic stem cells (ESCs), the absence of Pcgf1 did not affect the self-renewal of ESCs, but significantly affected the differentiation ability of ESCs, thus inhibiting the differentiation of ESCs into mesoderm and ectoderm. RNA-seq and GO analysis showed that Pcgf1 may activate genes related to mesoderm differentiation (Yan et al., 2017). In our study, we used zebrafish model to detect changes in a series of neural markers, such as Sox2, Otx2, and Ngn1, in neural induction process, and found that the expression of these markers was activated abnormally in the Pcgf1 MO group. Meanwhile, the BrdU-positive cells were decreased, the expression of PCNA was reduced, and the expression of the CDK inhibitors p21 and p57 were increased during NSC self-renewal stage after injection of the Pcgf1 MO. These results indicated that the inhibition of Pcgf1 expression during early embryonic development will affect the neural induction process, leading to abnormal activation of genes related to neural development, and then weakening the self-renewal and proliferation of NSCs, and finally cause the damage of telencephalon development. It is suggested that Pcgf1 plays an important role in neural tube development and telencephalon formation, which may affect both the neural induction stage and the self-renewal of NSCs. The discovery is a breakthrough point in the study of the function of such genes *in vivo*.

P19 cells are derived from an embryo-derived teratocarcinoma, and these cells can differentiate into each of the three germ layers (Boheler et al., 2002; Morikawa et al., 2013). RA-induced P19 cells have been used to study the differentiation of the three germ layers (Chen et al., 2014b). In our study, we used this model to study the effect and mechanism of Pcgf1 on the neural induction process. Our RNA-seq analysis showed that 1,502 target genes were upregulated in Pcgf1-KD P19 cells compared to wild type, whereas 243 target genes were downregulated in all 1,745 target genes with more than 2-fold altered expression levels in Pcgf1-KD P19 cells. GO analysis revealed that not only the signaling pathways but also the histone demethylase was affected by Pcgf1. Our results indicated that Pcgf1 may regulate neural induction process through dual mechanisms. Moreover, GO analysis further revealed that Pcgf1 could maintain the pluripotency of cells through regulating genes related to pluripotency maintenance.

The PcG protein family has always been considered as a class of proteins that inhibit the transcription of target genes at the level of chromatin. A previous study showed that Pcgf1 promotes monoubiquitinylation of histone H2A *in vivo* and *in vitro*, and knockdown of Pcgf1 reduces H2A ubiquitinylation in Hela cells (Wu et al., 2008). In our study, the RNA-seq data showed that the expression levels of histone demethylases (Kdm5a, Kdm5d, and Kdm7a) were upregulated after Pcgf1 knocked down. Meanwhile, the global levels of H3K27me3 and H3K4me3 were both decreased. This suggests that Pcgf1-mediating gene expression in RA-induced P19 cells might be histone methylation-dependent, and we correlated the pre-activation of neural induction in Pcgf1-KD P19 cells with the reduced abundance of repressive H3K27me3 and active H3K4me3 marks at the neural/pluripotency markers. The results of ChIP-qPCR *in vivo* further confirmed that the levels of H3K27me3 at the promoters of Ngn1/Otx2, and the levels of H3K4me3 at the promoters of Pou5f3/Nanog, were decreased after injecting Pcgf1 MO to the embryos. There are a variety of evidences supporting the role of PcG in transcriptional activation (Morey et al., 2015; Piunti and Shilatifard, 2016). The Pcgf5–PRC1–AUTS2 complex is involved in gene activation by the transcriptional co-activator P300 (Gao et al., 2014). Our data suggest that Pcgf1 may function as an activator and a repressor on different genes at the same time. However, Ezh2^−/−^ mutants with reduced H3K27me3 did not reveal severe brain phenotypes, suggesting that the role of Pcgf1 may be involved in other related mechanisms, which needs further study (San et al., 2016; Dupret et al., 2017). However, the findings in transgenic mice that Ezh2 ablation promoted ectopic expression of a forebrain transcriptional program involving derepression of the forebrain determinants revealed that Ezh2/H3K27me3-mediated gene repression is required for appropriate brain growth (Zemke et al., 2015).

## Conclusions

Taken together, we first discovered the crucial role of Pcgf1 in early neural tube development especially in neural induction phase in the embryo and established the regulatory network of transcription factors and epigenetic factors to neural induction, which laid a certain foundation for further exploring the function of other members during early neural tube development. From the perspective of disease treatment, this study provides a good basis for further revealing the role of the Pcgf family in the abnormal development of the neural tube and provides new ideas and new clues for the clinical treatment of abnormal neural tube closure.

## Data Availability Statement

The raw data supporting the conclusions of this article will be made available by the authors, without undue reservation.

## Ethics Statement

The animal study was reviewed and approved by the National Institutes of Health Guide for the Care and Use of Laboratory, and the Institutional Animal Care and Use Committees of Shandong University. Written informed consent was obtained from the owners for the participation of their animals in this study.

## Author Contributions

XinL performed most of the experiments and interpreted the data. GJ and JZ were involved in the *in vivo* experiments and assembly of data. JD, XiaL, WS, and YH contributed to the cell culture experiments and data analysis. WZ interpreted the data and co-wrote the manuscript with AH. AH provided critical input to the overall research design and final approval of the manuscript. All authors contributed to the article and approved the submitted version.

## Conflict of Interest

The authors declare that the research was conducted in the absence of any commercial or financial relationships that could be construed as a potential conflict of interest.

## Abbreviations

PRC1: polycomb repressive complex 1
NSCs: neural stem cells
MO: morpholino
CDK: cyclin-dependent kinase
RA: retinoic acid.
