# Supplementary figures and images for: Pcgf1 Regulates Early Neural Tube Development Through Histone Methylation in Zebrafish
Source: Front Cell Dev Biol. 2021 Jan 26;8:581636. doi: 10.3389/fcell.2020.581636 (PMC7870693; doi:10.3389/fcell.2020.581636)

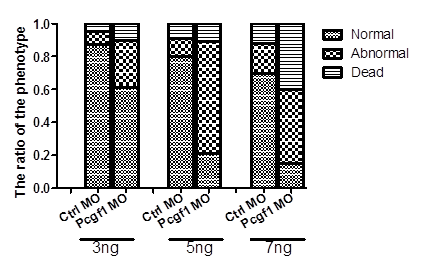

Supplement: Supplementary file 1 [file Image_1.TIF]
